# Notes and Comments

**Published:** 1899-12

**Authors:** 


### Notes and Comments.[1]

Cocaine and Eucaine.—In response to a request for a method for distinguishing eucaine from cocaine, we reprint the following taken from *L'Odontalgique.*

"The excessive solubility of hydrochlorate of cocaine permits its being distinguished from hydrochlorate of eucaine, for eucaine is soluble one part in nine of water, while cocaine is soluble in less than its own weight of water. To detect eucaine that may be

[1] The assistant editor solicits contributions for this department,—new methods, new remedies and formulas, or any short practical note which may prove of value to the practitioner or student. Address 1338 Walnut Street, Philadelphia.

fraudulently added to cocaine, because of its costing less, Vulpius states that by dissolving ten grains of the suspected salt in fifty cubic centimetres of water, and then adding two drops of aqua ammonia, if the cocaine is free from eucaine the liquid will remain clear, even though a few crystals may be deposited, whilst if eucaine is present the solution becomes cloudy or milky in appearance."

---

Durability of Cement Fillings.—In reference to the durability of cement fillings, the *Pharmaceutical Journal* says the durability of the filling is greatly increased by condensing it thoroughly by pressure while it is setting. This is best done with a smooth, round-headed hand burnisher, kneading it, so to speak, into the cavity. The rubber dam should be used, and as much care taken in shaping and finishing the cavity as for a gold filling. A stopping so treated with melted parffin flowed over, using the hot-air syringe to keep it melted while it soaks into the cement, will be found (the conditions of the mouth being favorable) to last for many years, even in the most exposed positions. It is as remarkable as it is true that the walls of carefully prepared cavities that have been filled with cement seldom show any signs of recurring decay, although the stopping may be worn away considerably below the enamel edge.

---

Fads and Faddists.—Our lamented *American Dental Weekly* said some time ago, in speaking of fads, " It is right hard to retain an equipoise during such times, and few, comparatively, do so. Almost every individual dentist has a fad of some kind, and at times one or more are brought prominently before the profession. Prominent men, and journals as well, will take hold of them, and for a while scarcely anything else is heard but the fadded thing. Sometimes good results come from fads. If in no other way, they cause some people to think, which act will cause the promulgation of an idea or thought that leads to good and lasting results.

" Again, fads, when they have run their course, teach by experience, and such lessons are not easily forgotten; but the investigation put forth by some who are carried with the whirl and stranded on the shore of despair often points the way out of a danger to others, and to a more reasonable procedure. As an illustration, the putting into active and indiscriminate practice, a few years back, by the medical profession, that part of the Mosaic law relating to circumcision. It was merely a fad for a while, but to the thoughtful and reasoning, the benefits in a majority of cases changed a fad to an absolute necessity.

"To do something new often leads the young and inexperienced into the fadding of methods which often are valuable, but which require mature judgment for their proper execution.

"How perfectly familiar the copper-amalgam fad is to the reader! It required the greatest amount of conservatism to withstand the onslaughts of this, the fad of fads. Then there came the 'Herbst method,' when manufacturers put thousands of dollars into the new instruments that were required to practise this fad. The journals teemed with it, the societies gloated over it; only occasionally would be heard the well-poised warning against it. Everything seemed to be pell-mell, all going Herbstward. Where now are the method and the instruments?

"Then came cataphoresis. Factories were putting out the best machines all over the country, every man who had used it rushed into print and before societies. Now one of the leaders says he thought he knew something about it, but finds, after *experience*, that he does not, and declares that the perfect machine has yet to be made.

"The results of cataphoresis are sometimes wonderful, and sometimes disastrous. It requires the thinking, conservative man—the one who neither rushes into print nor before the society—to get out of it what there is in it."

---

LORETIN.—Dr. Esch, of Eisenach, reports that loretin fulfils all the properties of iodoform. It is superior to the latter owing to its being odorless, does not cause skin eruptions nor iodism,—three great advantages which should be highly valued by the practitioner. He also reports excellent results with loretin-bismuth in ulcers of the leg, not only those which were due to specific causes, but also those of varicose origin. He used it in about thirty cases in the following manner: The floor of the ulcer should be first wiped out with sterilized cotton-wool, after which it should be freely dusted with loretin-bismuth, and sterilized cotton-wool applied; the leg should be elevated and a bandage loosely applied. As the cotton-wool becomes soaked with the secretion of the wound, loretin-bismuth should be dusted on and around it, and fresh portions of cotton-wool placed on the old one; or a completely fresh dressing can be applied. Within two or three weeks he was able to heal up a few dozen ulcers of the leg which had existed for some years, some of which were considerable in extent.—*Therapist.*

CATAPHORESIS AND ARSENIC.—Dr. Fletcher, in the *Dental Digest*, says, "A dentist in this city made an application of arsenic in the usual manner, and at the next sitting attempted to remove the pulp, but found it highly sensitive. To hasten matters he applied cocaine with the current and removed the pulp painlessly, but at the next sitting he found the arsenic had been inducted into tissues beyond the tooth. Here was the devil to pay and no funds. Don't say he should have known better; any one might have done the same thing thoughtlessly."

